# Omicron BA.1 Mutations in SARS-CoV-2 Spike Lead to Reduced T-Cell Response in Vaccinated and Convalescent Individuals

**DOI:** 10.3390/v14071570

**Published:** 2022-07-19

**Authors:** Maarten E. Emmelot, Martijn Vos, Mardi C. Boer, Nynke Y. Rots, Jelle de Wit, Cécile A. C. M. van Els, Patricia Kaaijk

**Affiliations:** 1 Centre for Infectious Disease Control, National Institute for Public Health and the Environment (RIVM), 3721 MA Bilthoven, The Netherlands; maarten.emmelot@rivm.nl (M.E.E.); martijn.vos.02@rivm.nl (M.V.); mardi.boer@rivm.nl (M.C.B.); nynke.rots@rivm.nl (N.Y.R.); jelle.de.wit@rivm.nl (J.d.W.); cecile.van.els@rivm.nl (C.A.C.M.v.E.); 2 Faculty of Veterinary Medicine, Utrecht University, 3584 CL Utrecht, The Netherlands

**Keywords:** SARS-CoV-2, Omicron BA.1 variant, mutations, T-cell response, vaccination, natural infection, cross-reactivity, immune escape, CD4^+^ T-cell epitopes, HLA motif prediction

## Abstract

Omicron BA.1 variant can readily infect people with vaccine-induced or naturally acquired SARS-CoV-2 immunity facilitated by escape from neutralizing antibodies. In contrast, T-cell reactivity against the Omicron BA.1 variant seems relatively well preserved. Here, we studied the preexisting T cells elicited by either vaccination with the mRNA-based BNT162b2 vaccine or by natural infection with ancestral SARS-CoV-2 for their cross-reactive potential to 20 selected CD4^+^ T-cell epitopes of spike-protein-harboring Omicron BA.1 mutations. Although the overall memory CD4^+^ T-cell responses primed by the ancestral spike protein was still preserved generally, we show here that there is also a clear loss of memory CD4^+^ T-cell cross-reactivity to immunodominant epitopes across the spike protein due to Omicron BA.1 mutations. Complete or partial loss of preexisting T-cell responsiveness was observed against 60% of 20 nonconserved CD4^+^ T-cell epitopes predicted to be presented by a broad set of common HLA class II alleles. Monitoring such mutations in circulating strains helps predict which virus variants may escape previously induced cellular immunity and could be of concern.

## 1. Introduction

The severe acute respiratory syndrome coronavirus-2 (SARS-CoV-2) Omicron BA.1 variant (B.1.1.529) was first identified in Botswana and South Africa in early November 2021 and was later defined as a new variant of concern (VOC) by the World Health Organization on 26 November 2021 [1]. Since then, this variant has rapidly spread to many countries. The Omicron BA.1 variant is associated with enhanced transmissibility and largely escapes the neutralizing antibodies elicited after previous vaccination or infection [2,3,4,5,6,7]. The current commonly used vaccines that induce protective immune responses against severe coronavirus disease-19 (COVID-19) rely on eliciting immunity against the spike protein of ancestral SARS-CoV-2 [8,9,10,11,12]. The Omicron BA.1 variant contains at least 30 mutations in the spike protein, which mediate escape from vaccine-induced neutralizing antibodies. However, additional booster vaccine doses have shown to partially compensate for the diminished neutralization [2,3,5,13].

T cells are important in containing viral replication and ensuring viral clearance [14,15]. Early and robust SARS-CoV-2-specific T-cell responses have been associated with limiting the severity of COVID-19 [15,16,17]. Vaccine-induced or naturally acquired memory T-cell immunity contribute to remarkable protection against hospitalization or death due to COVID-19 [17,18,19]. Additionally, T-cell responses have shown to be important in controlling SARS-CoV-2 infection in patients with immune disorders, causing impaired antibody responses [20]. Moreover, in contrast to antibody mediated immunity, T-cell responses to the Omicron BA.1 variant have been shown to be more preserved [13,19,21,22]. T cells induced after previous SARS-CoV-2 vaccination or infection may therefore contribute to limiting COVID-19 severity after infection with Omicron BA.1 virus that escapes neutralizing antibodies [23]. However, albeit relatively preserved, T-cell responses to the Omicron spike protein are reported to be, on average, 10–30% decreased compared with the spike protein of the ancestral SARS-CoV-2 strain [13,19,21,22,24].

The various studies conducted to investigate the T-cell cross-recognition of specific epitopes of the SARS-CoV-2 variants have been mainly limited to sequence comparison and subsequent in silico prediction of peptide–HLA binding [25,26]. In our proof-of-concept study, we assessed the functional cross-reactivity of T cells from prior-vaccinated or infected subjects against selected spike epitopes carrying Omicron BA.1 mutation(s). For this purpose, we focused on CD4^+^ T-cell epitopes of spike protein, as CD4^+^ T cells have shown to dominate the spike-specific T-cell response [18,27,28]. To cover a broad HLA class II restriction for the general population, bioinformatics-guided identification of promiscuous helper epitope candidates was applied. We selected 20 spike epitopes of ancestral SARS-CoV-2 predicted to be restricted by multiple common HLA-DR, -DP, and -DQ alleles, and harboring Omicron BA.1 mutations [29].

The present study aims to understand the impact of Omicron BA.1 mutations in a panel of (immunodominant) epitopes on the cross-reactivity of preexisting T cells. We focused on preexisting T cells elicited by either vaccination with the mRNA-based BNT162b2 vaccine or by natural infection with ancestral SARS-CoV-2. Identification of mutations in the spike protein that can evade vaccine-induced or naturally acquired T cell memory, can help to efficiently monitor immune escape mutants and estimate their potential impact on protection against COVID-19.

## 2. Materials and Methods

### 2.1. Clinical Samples

Blood samples used were collected in two clinical studies, a SARS-CoV-2 vaccination cohort study (samples taken before October 2021, thus before Omicron variants were identified), and a previously described SARS-CoV-2 infection cohort study (2020) [28,30]. The protocol for the SARS-CoV-2 vaccination study was approved by the Medical–Ethical Review Committee (MERC) of University Medical Center Utrecht; EudraCT number: 2021-001357-31. The protocol for the SARS-CoV-2 infection study was approved by the Medical–Ethical Review Committee (MERC) of University Medical Center Utrecht; Netherlands Trial Register (NTR) number: NL9850 (https://trialsearch.who.int/, accessed on 5 June 2022). Written informed consent was received from all subjects prior to study-specific procedures. All trial-related activities were conducted according to Good Clinical Practice, including the provisions of the Declaration of Helsinki.

From the vaccination cohort, blood samples were used from 10 healthy adult subjects (5 males and 5 females) with a mean age of 29 years (range 23–39 years). Participants received two doses of the mRNA-based BNT162b2 vaccine, with a median interval of 35 days (range 35–37 days). According to a questionnaire, these vaccinated subjects had not been previously infected with SARS-CoV-2 and also tested negative for the presence of antibodies against SARS-CoV-2 nucleocapsid protein [31], and negative for presence of anti-S1 antibodies at pre-vaccination. Blood samples were taken 28 days (range 26–42 days) after their second vaccine dose. From the infection cohort, blood samples were used from PCR-confirmed SARS-CoV-2-infected subjects that were taken 6–8 weeks post symptom onset. Subjects were infected in the beginning of the COVID-19 pandemic (March–May 2020), and thus prior to the emergence of the Omicron BA.1 variant. Blood samples were used from 6 convalescent adult subjects (3 male and 3 female) with mean age of 38 years (range 18–51 years). Although the infection cohort was initially set up to study SARS-CoV-2 infection rates within households, all included 6 subjects were from different households.

### 2.2. Peripheral Blood Mononuclear Cells

Peripheral blood mononuclear cells (PBMCs) were isolated from heparinized blood samples by centrifugation on a Ficoll-Hypaque gradient (Pharmacia Biotech, Kalamazoo, MI, USA) and cryopreserved at −135 °C until use.

### 2.3. Prediction of CD4^+^ T-Cell Epitope Candidates

For the spike protein of the Omicron BA.1 variant the following mutations, deletions and insert in the amino acid sequences of the spike protein of the D614G wild-type (WT) reference SARS-CoV-2 (UniProtKB: P0DTC2) were considered [32]: A67V, Δ69–70, T95I, G142D, Δ143–145, Δ211, L212I, ins214EPE, G339D, S371L, S373P, S375F, K417N, N440K, G446S, S477N, T478K, E484A, Q493R, G496S, Q498R, N501Y, Y505H, T547K, D614G, H655Y, N679K, P681H, N764K, D796Y, N856K, Q954H, L981F. Notably, Omicron BA.1 mutation N969K was not included in the study. This mutation is therefore missing in the peptides used. The following 19 common HLA-II types were selected for HLA Class II motif prediction (based on a previous described panel [29]): HLA-DRB1*0101, 0301, 0401, 0405, 0701, 0802, 0901, 1101, 1201, 1302, and 1501; HLA-DRB3*0101 and 0202; HLA-DRB4*0101; HLA-DQA1*0501/HLA-DQB1*0301; HLA-DQA1*0301/HLA-DQB1*0302; HLA-DQA1*0102/HLA-DQB1*0602; HLA-DPA1*0103/HLA-DPB1*0401; HLA-DPA1*0103/HLA-DPB1*0402.

NetMHCIIpan-4.0 (accessed: 13 December 2021) [33] was used to predict the binding affinity as well as the likelihood to be naturally presented via the selected 19 different HLA-II alleles. As input, all possible 15-mer peptides spanning the whole spike protein sequence of D614G WT reference strain (UniProtKB: P0DTC2) were used. For this purpose, both the % rank score of the binding affinity prediction (BA data) and the % rank score for likelihood of a peptide to be naturally presented (EL data) for each of the selected HLA-II alleles were considered. The % rank scores normalizes prediction score by comparing to prediction of a set of 100,000 random natural peptides [33]. Criteria for peptide selection were: % rank score < 2.00 for BA or EL data of at least one HLA-II allele, and % rank score of BA or EL data between 2.01 and 10.00 for at least 5 different HLA-II alleles.

### 2.4. Peptide Synthesis and Peptide Pools Preparation

Custom-ordered 15-mer peptides representing the 20 selected individual CD4^+^ T-cell epitope candidates of the spike protein of the D614G WT reference strain and their corresponding Omicron BA.1 counterparts (Table 1) were synthesized for single use (JPT, Berlin, Germany). In addition, two customized peptide pools were made, one pool representing the 20 selected WT epitope candidates (“WT CD4^+^ pool”), and one peptide pool consisting of the 20 Omicron BA.1 epitope counterparts (“Omicron CD4^+^ pool”) (JPT, Berlin, Germany). Two commercial pools of overlapping synthetic peptides (15-mers, with 11 overlap) consisting of, respectively, 316 and 315 peptides, one covering whole spike protein (S1/S2) of the D614G WT SARS-CoV-2 strain (“WT S1/S2 pool”) and one covering the whole spike protein of the Omicron BA.1 variant (“Omicron S1/S2 pool”) were purchased (JPT, Berlin, Germany). Peptides were resuspended in dimethyl sulfoxide (DMSO). Subsequently, customized single peptides and CD4^+^ pools were diluted with PBS to a stock concentration of 50 µM, while the spike S1/S2 pools were diluted with PBS to a stock concentration of 75 µM. The final DMSO concentrations for peptide stimulations was 0.1–0.3% for peptide pools and 0.02% for single peptides.

### 2.5. Preparation of T-Cell Lines

In order to generate T-cell lines, PBMCs of all 10 vaccinated and all 6 convalescent subjects were thawed and cultured for two weeks in AIM-V medium (12055–083, Gibco, Waltham, MA, USA) supplemented with 2% human AB serum (H6914, Sigma, Kawasaki, Kanagawa) in the presence of “WT CD4^+^ pool” at 0.5 µM/peptide. IL-2 (5 ng/mL, 130–097–743, Miltenyi Biotec, Bergisch Gladbach, Germany) was added, and, if necessary, wells were split on day 4, 7, and 11. On day 14, the T-cell lines were tested by ELISPOT or flow cytometry. Remaining cells were frozen (≥1.5 × 10^6^ per vial).

### 2.6. IFN-ɣ ELISPOT

Multiscreen filtration ELISPOT plates (Millipore (Burlington, MA, USA), Merck (Kenilworth, NJ, USA), MSIPS4510) were prewetted with 35% ethanol for ≤1 min and washed with sterile water. Plates were coated overnight (4 °C) with 5 μg/mL antihuman IFN-ɣ antibodies (1-D1K, Mabtech, Stockholm, Sweden), washed with PBS, and then blocked for at least 30 min with AIM-V medium (Lonza, Basel, Switzerland) with 2% human serum (Sigma). PBMCs or T-cell lines, 2 × 10^5^ cells/well and 0.5 × 10^5^ cells/well, respectively, were incubated with the peptide pools covering whole spike protein, i.e., “WT S1/S2 pool” or “Omicron S1/S2 pool” (0.5 μM/peptide) or with the 20 selected epitope candidates, i.e., “WT CD4^+^ pool” or “Omicron CD4^+^ pool” (1 μM/peptide). In addition, T-cell lines were incubated with the individual peptides (1 μM). Cells were incubated for 20 h, 37 °C, 5% CO_2_ in 100 μL AIM-V with 2% human serum. DMSO and PHA (1 µg/mL; Sigma) were negative and positive controls, respectively. Subsequently, plates were washed and incubated for 1 h with 1 μg/mL antihuman IFN-ɣ-detection biotinylated antibody (7-B6–1, Mabtech) in PBS-0.5% FBS. Plates were washed and incubated with Streptavidin–poly–horseradish peroxidase (HRP) (Mabtech) in PBS-0.5% FBS for 1 h. After washing, plates were developed with 3,3′,5,5′-Tetramethylbenzidine (TMB) substrate (Mabtech). Spots were analyzed with CTL software. The number of spots from negative DMSO controls was subtracted from total spot numbers induced by antigen-specific stimulation; more than 5 spots, after background subtraction, were considered to indicate a positive result.

### 2.7. Flow Cytometry-Based T-Cell Assays

T-cell lines were analyzed after stimulation with “WT CD4^+^ pool” or “Omicron CD4^+^ pool” (1 μM/peptide) for 6 h. During the last 5 h, a mixture of Brefeldin A and Monensin (Biolegend, San Diego, CA, USA) was added. Cells were stained for antihuman CD3 (clone HIT3A; BioLegend), CD4 (clone SK3), and CD8 (clone RPAT8; both BD Biosciences, Franklin Lakes, NY, USA). After fixation and permeabilization, using FoxP3/Transcription Factor Staining Buffer Set (eBioscience, San Diego, CA, USA, Thermo Fisher Scientific, Waltham, MA, USA), cells were stained intracellularly for antihuman, CD154 (clone TRAP1; BD Bioscience), and cytokines: IFN-ɣ (clone 4S.B3; BD Bioscience), IL-2 (clone MQ1–17H12; Thermofisher), or TNF-α (clone Mab11; Thermofisher). Cells were acquired on a FACS Symphony A3 analyzer (BD) and analyzed using FlowJo (V10, Tree Star, Ashland, OR, USA). On average, 40,000 events were acquired; however, in the T-cell lines obtained from the convalescent subjects, the number of events was often somewhat lower (i.e., 8000 events).

### 2.8. Cytokine Release Assay

T-cell lines, at 0.5 × 10^5^ cells/well, were incubated for 24 h with the individual peptides (1 μM) in round-bottom 96-well plates. Cell-free culture supernatants were harvested from the stimulated T-cell lines and analyzed using a bead-based multiplex immunoassay (MIA), quantitating levels of IFN-ɣ, TNF-α, IL-2, IL-4, IL-5, IL-13, IL-10, IL-22, IL-6, IL-9, IL-17A, and IL-17F (LEGENDplex human Th cytokine panel, 741028; BioLegend) according to the manufacturer’s instructions and using FACSCanto II (BD). DMSO (an equimolar amount of DMSO as used for peptide stimulations) and PHA (Sigma, 1 µg/mL) were used as negative and positive controls, respectively. For analysis, the online cloud-based program, the LEGENDplex™ Data Analysis Software Suite, was used. Background signal from negative DMSO controls was subtracted from total concentration (pg/mL) per cytokine induced by antigen-specific stimulation. If the background signal was below the threshold for detection, the detection threshold concentration was subtracted from the measured cytokine concentration induced by specific stimulation.

### 2.9. Statistical Analysis

Statistical analyses were performed in Prism (version 9.3.1; GraphPad Software). The Wilcoxon signed-rank *T* test was used to compare responses of (paired) samples after stimulation with peptides of the WT reference strain versus the Omicron BA.1 variant. Mann–Whitney U test was performed to analyze differences between the groups of vaccinated versus convalescent subjects with respect to the T-cell response or ratio Omicron/WT of the T-cell response; *p* values less than 0.05 were considered statistically significant.

## 3. Results

### 3.1. Prediction and Selection of Broadly Reactive CD4^+^ T-Cell Epitopes of Spike-Protein-Containing Omicron Mutations

**Figure 1:**
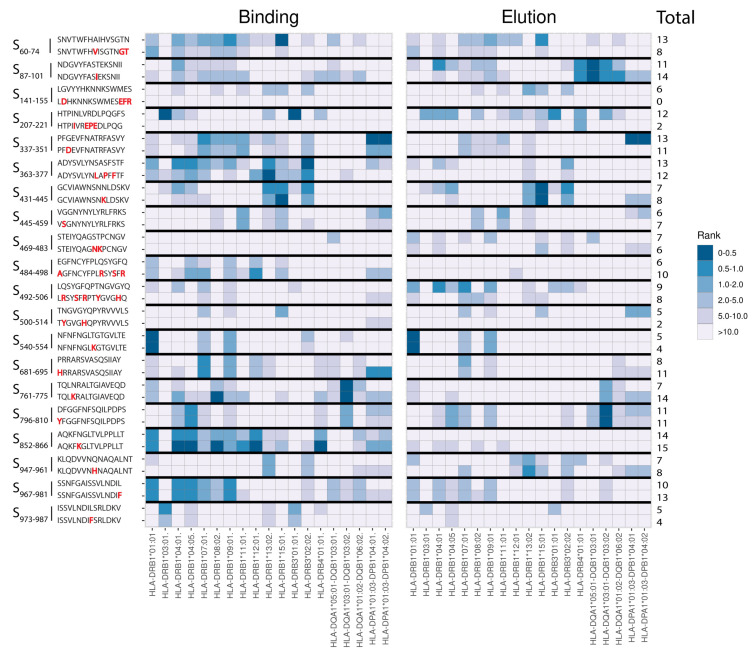
Heatmap showing predicted T cell immunogenicity scores of selected epitope candidates for multiple common HLA-II alleles. Predicted T cell immunogenicity scores of the CD4^+^ T-cell epitope candidates of the spike protein of the D614G wild-type (WT) SARS-CoV-2 strain were plotted next to the scores of the corresponding Omicron BA.1 variant peptides having single or multiple mutations (including insertions/deletions). Color scale in heat map indicates differences in predicted HLA-II binding affinity scores (**left panel**) or elution scores (**right panel**) to the various HLA-II alleles as depicted below. Peptides with lower rank scores (dark blue) represent strongly predicted T-cell epitopes. Each peptide pair is indicated as location of first and last amino acid position within WT spike protein (S), the WT spike sequence is presented on top and the Omicron BA.1 spike sequence below it. Differences in amino acid sequences between the Omicron BA.1 peptides compared with the epitope candidates of the WT ancestral spike are marked in red. On the right side of the figure, for each peptide the total number of HLA-II alleles is indicated that had a % rank score < 10.0 for either binding affinity prediction or elution % rank score < 10.0.

Shortly after identification of the Omicron BA.1 (B.1.1.529) variant, we performed a T-cell epitope screening to select highly promiscuous CD4^+^ T-cell epitope candidates of spike protein of the ancestral D614G SARS-CoV-2 as wild-type reference strain (WT spike), in which the Omicron BA.1 variant contains mutations. A total of 20 CD4^+^ T cell candidate epitopes (15-mers) of WT spike-containing Omicron BA.1 mutations were selected based on best prediction scores for 19 common HLA-II alleles using a bioinformatic tool [33] (Figure 1, Table 1). Interestingly, all 20 selected candidate epitopes matched epitope sequences with proven T-cell immunogenicity available in the IEDB database [34] (Supplementary Table S1). Next, we investigated whether Omicron BA.1 mutations in these 20 epitopes had an effect on prediction scores for the various HLA alleles. Remarkably, the corresponding Omicron BA.1 counterparts of the selected promiscuous CD4^+^ T-cell epitope candidates of WT spike generally showed reasonably good prediction scores for multiple HLA-II alleles as well (Figure 1).

### 3.2. Ex Vivo T-Cell Responses against Omicron BA.1 Whole Spike Protein Are Relatively Preserved in Most Prior-Vaccinated and Convalescent Individuals

Custom-made 15-mer peptides were synthesized, representing these 20 selected CD4^+^ T-cell epitopes of spike protein of the D614G WT reference strain and their corresponding sequences containing Omicron BA.1 mutations, in order to be used individually or as dedicated peptide pools (“WT CD4^+^ pool” and “Omicron CD4^+^ pool”, respectively) in functional T-cell assays (Table 1).

Subsequently, we performed an ELISPOT to enumerate spike-specific IFN-ɣ^+^-producing cells in the peripheral blood of vaccinated or convalescent individuals. For this purpose, PBMCs were stimulated either with pools of overlapping peptides (15-mers, with 11 overlap) covering the whole spike protein (S1/S2) of the D614G WT SARS-CoV-2 strain (“WT S1/S2”) or the whole Omicron BA.1 variant spike protein (“Omicron S1/S2”), or with the respective “WT CD4^+^ pool” or “Omicron CD4^+^ pool”. In the 10 vaccinated subjects (23–39 years), sampled 28 days post-primary COVID-19 mRNA vaccination and with no evidence of previous SARS-CoV-2 infection, frequencies of IFN-ɣ^+^ T cells reactive to the “Omicron S1/S2 pool” were slightly reduced compared with the “WT S1/S2 pool” (respectively, 22 median spot-forming units (SFU)/2.10^5^ PBMCs) versus 26 SFU/2.10^5^ PBMCs; *p* = 0.018). In the 6 convalescent subjects (18–51 years), samples collected at 6–8 weeks post symptom onset after infection with ancestral SARS-CoV-2 (March–May 2020), frequencies of IFN-ɣ^+^ T cells reactive to “WT S1/S2 pool” were in a similar magnitude as vaccinated individuals. However, frequencies of IFN-ɣ^+^ T cells reactive to “Omicron S1/S2 pool” were significantly lower in PBMCs of vaccinees than in PBMCs of convalescent subjects (*p* = 0.031; median SFU/2.10^5^ resp. 22 versus 47).

**Figure 2:**
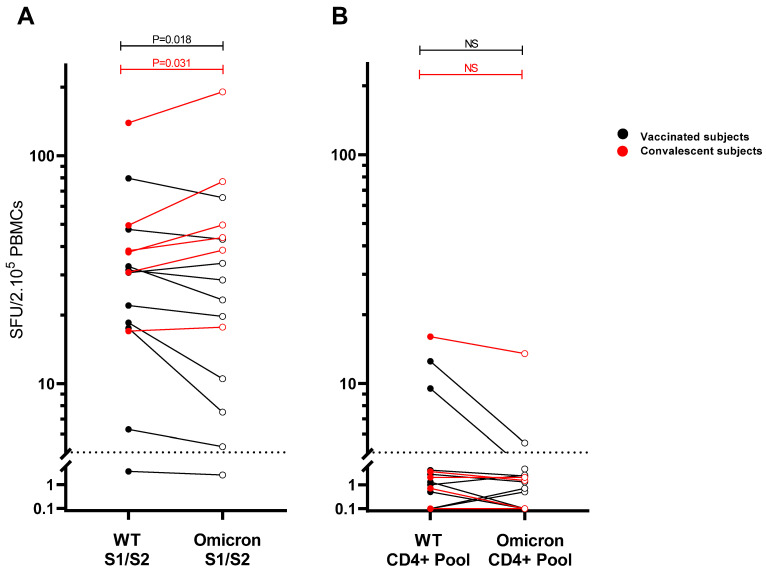
Ex vivo T-cell responses against Omicron BA.1 whole spike protein are relatively preserved in most prior-vaccinated and convalescent individuals. The SARS-CoV-2-specific IFN-ɣ^+^ T-cell response was measured by ELISPOT assay. PBMCs of vaccinated (*n* = 10; black dots/lines) and convalescent individuals (*n* = 6; red dots/lines) were directly stimulated with (**A**) a pool of overlapping peptides for the entire spike protein (S1 + S2) corresponding to the D614G wild-type (WT) strain versus the Omicron BA.1 variant or with (**B**) a pool of selected CD4^+^ T-cell epitope candidates from WT strain versus the corresponding sequences of the Omicron BA.1 variant. Each pair of dots connected with line represents SARS-CoV-2-specific IFN-ɣ^+^ T-cell response of one subject responding to WT (closed dots) versus Omicron BA.1 peptides (open dots). Dotted line indicates threshold for IFN-ɣ-positive responses. Pairwise comparison of T-cell responses to WT and Omicron BA.1 peptide pools assessed by Wilcoxon signed-rank *T* test revealed a significant difference in response in vaccinees (reduction in response to overlapping S1/S2 Omicron peptide pool; *p* = 0.018) and convalescent subjects (increased response to S1/S2 Omicron peptide pool; *p* = 0.031). SFU—spot forming units.

Furthermore, and quite unexpectedly, in the convalescent subjects the cross-reactive T-cell response against the overlapping “Omicron S1/S2 pool” showed a slight increase compared with the “WT S1/S2 pool” (38 versus 47 SFU/2.10^5^ PBMCs; *p* = 0.031) (Figure 2A).

When zooming in on the spike-specific epitopes that varied between WT and Omicron, measured frequencies of IFN-ɣ^+^ T cells were low or undetectable in most subjects, and therefore inconclusive. However, considering only the three individuals that showed an IFN-ɣ^+^ response upon stimulation with the “WT CD4^+^ pool”, especially the two vaccinees did show a reduced response to the “Omicron CD4^+^ pool” (Figure 2B).

### 3.3. Reduced Cross-Reactivity of Spike-Epitope-Specific T-Cell Lines Due to Mutations in the Omicron BA.1 Variant in Prior-Vaccinated and Convalescent Subjects

**Figure 3:**
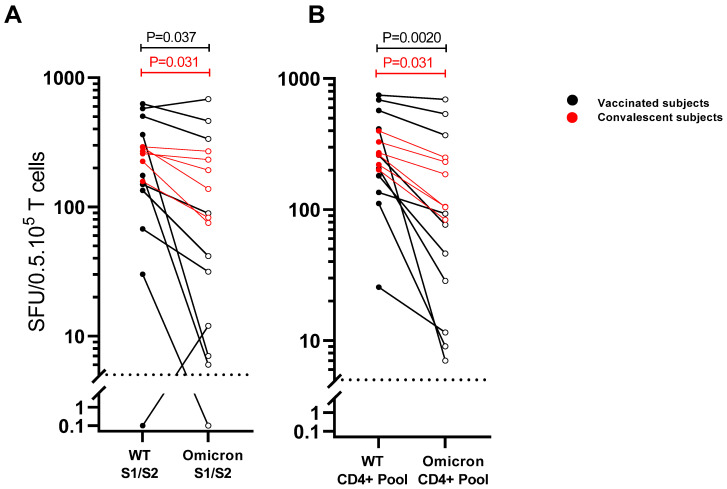
Reduced cross-reactivity of spike-epitope-specific T-cell lines due to mutations in the Omicron BA.1 variant in prior-vaccinated and convalescent subjects. Recognition of peptide pools of spike protein of D614G wild-type (WT) SARS-CoV-2 strain versus the BA.1 Omicron BA.1 variant by polyclonal-epitope-specific T-cell lines in IFN-ɣ ELISPOT assay. T-cell lines were generated by 14-day in vitro stimulation of PBMCs from vaccinated (black dots/lines) and convalescent individuals (red dots/lines) with a pool of selected CD4^+^ T-cell epitope candidates from the WT strain (WT CD4^+^ pool) in the presence of IL-2. Subsequently, cells were stimulated for 24 h with (**A**) a pool of overlapping peptides for the entire spike protein (S1 + S2) corresponding to the WT strain versus the Omicron BA.1 variant or with (**B**) the “WT CD4^+^ pool” versus the corresponding sequences of the Omicron BA.1 variant. Each pair of dots connected with line represents SARS-CoV-2-specific IFN-ɣ^+^ T-cell response of one subject responding to WT versus Omicron BA.1 peptides (closed dots) versus Omicron BA.1 peptides (open dots). Dotted line indicates threshold for IFN-ɣ-positive responses. Pairwise comparison of T-cell responses to WT and Omicron BA.1 peptide pools assessed by Wilcoxon signed-rank *T* test revealed a significant reduction in response to both Omicron peptide pools in both vaccinees and convalescent subjects. SFU—spot forming units.

Next, we employed antigen-specific T-cell enrichment to be able to further study and characterize the cross-reactive potential of the T cells reactive to spike epitopes harboring Omicron BA.1 mutations. For this purpose, T-cell lines were generated from each of the vaccinated and infected subjects by 14-day in vitro stimulation of PBMCs with the “WT CD4^+^ pool”. In the group of vaccinated subjects, a significant reduction in frequencies of SARS-CoV-2-specific IFN-ɣ^+^ T cells was observed against the “Omicron S1/S2 pool” compared with the “WT S1/S2 pool” spanning the entire respective spike proteins (37 versus 162 SFU/2.10^5^ PBMCs; *p* = 0.037). Additionally, in the convalescent group, SARS-CoV-2-specific IFN-ɣ^+^ T-cell frequencies were lower after stimulation with the “Omicron S1/S2 pool” compared with the “WT S1/S2 pool” (165 versus 264 SFU/2.10^5^ PBMCs; *p* = 0.031) (Figure 3A). In line with these findings, the frequencies of SARS-CoV-2-specific IFN-ɣ^+^ T cells of prior-vaccinated and convalescent individuals were also significantly reduced after stimulation with the “Omicron CD4^+^ pool” compared with the “WT CD4^+^ pool” (respectively, 61 versus 232 (vaccinees) and 146 versus 266 SFU/2.10^5^ PBMCs (convalescent subjects)) (Figure 3B).

### 3.4. In-Depth Characterization of Reduced Cross-Reactivity of Spike-Specific T-Cell Lines to Omicron BA.1 Variant

**Figure 4:**
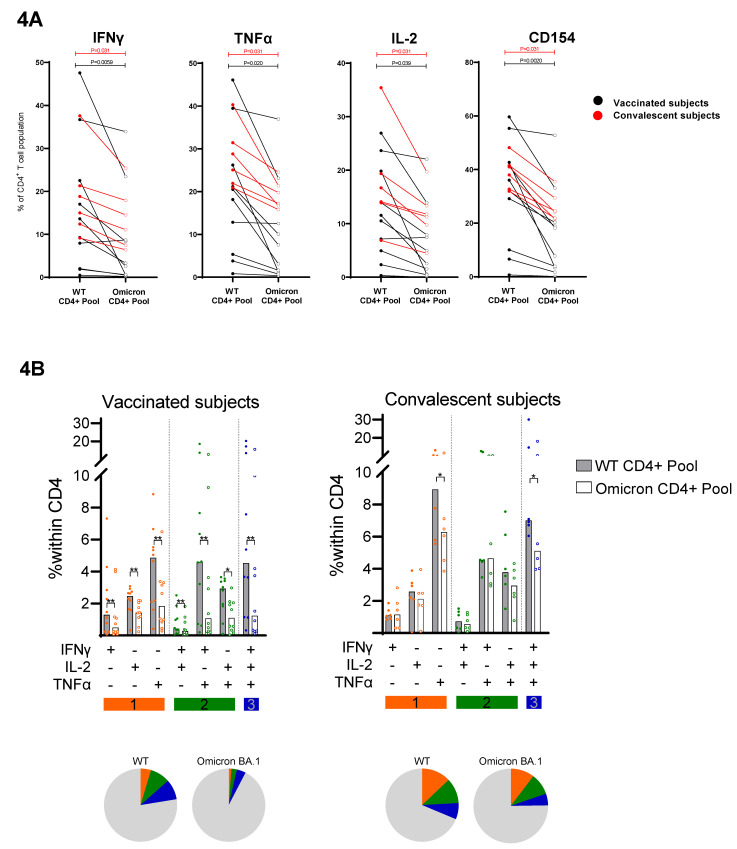
In-depth characterization of reduced cross-reactivity of spike-specific T-cell lines to Omicron BA.1 variant. Polyclonal-epitope-specific T-cell lines were analyzed for functional marker expression by flow cytometry after stimulation with peptide pools of spike protein of D614G wild-type (WT) strain versus Omicron BA.1 variant. (**A**) T-cell lines were generated by 14-day in vitro stimulation of PBMCs from vaccinated (black dots/lines) and convalescent subjects (red dots/lines) with a pool of selected CD4^+^ T-cell epitope candidates from the WT strain (“WT CD4^+^ pool”). Subsequently, cells were stimulated for 6 h with the “WT CD4^+^ pool” or the corresponding sequences of the Omicron BA.1 variant. Each pair of dots connected with line represents the percentage of SARS-CoV-2-specific CD4^+^ T cells expressing the functional marker, i.e., IFN-ɣ, TNF-α, IL-2 and CD154 of one subject responding to WT (closed dots) versus Omicron CD4^+^ pools (open dots). Pairwise comparison of T-cell responses to WT CD4^+^ pool and Omicron CD4^+^ pool assessed by Wilcoxon signed-rank *T* test revealed a significant reduction in response to the Omicron CD4^+^ pool in both vaccinees and convalescent subjects. (**B**) Dot plots showing the median proportion of CD4^+^ T cells secreting one, two, or three different cytokines in vaccinees (left panel) and convalescent subjects (right panel). Pie chart also shows the median proportion of CD4^+^ T cells secreting no, one, two, or three different cytokines. Orange color indicates secretion of one cytokine; green color indicates simultaneous secretion of two cytokines; blue color indicates simultaneous secretion of IFN-ɣ, TNF-α, and IL-2; grey color indicates no cytokine secretion. Pairwise comparison of T-cell responses to WT CD4^+^ pool and Omicron CD4^+^ pool assessed by Wilcoxon signed-rank *T* test revealed a significant reduction in single or polyfunctional cytokine response to Omicron CD4^+^ pool in both vaccinees and convalescent subjects, as indicated with asterisk. * *p* < 0.05, ** *p* < 0.01.

For more in-depth functional characterization of the WT versus Omicron-spike-specific T-cell populations, propagated T-cell lines of all subjects were restimulated with either the “WT CD4^+^ pool” or the “Omicron CD4^+^ pool” and analyzed by flow cytometry. In the T-cell lines of the vaccinees, high frequencies of SARS-CoV-2-specific CD4^+^ T cells producing IFN-ɣ, TNF-α, and/or IL-2 were measured by intracellular cytokine staining (ICS) after stimulation with the “WT CD4^+^ pool”. In line with the IFN-ɣ ELISPOT results, percentages of cytokine positive cells were significantly lower upon restimulation of the T-cell lines with the “Omicron CD4^+^ pool” (median 11% versus 2.9% IFN-ɣ^+^ of the CD4^+^ T-cell population (*p* = 0.0059); 19% versus 5.5% TNF-α^+^ (*p* = 0.039) and 11% versus 3.8% IL-2^+^ (*p* = 0.020)) (Figure 4A). In convalescent subjects, a similar pattern was observed; cytokine production was reduced in “WT CD4^+^ pool” versus “Omicron CD4^+^ pool” restimulated T-cell lines (17% versus 13% IFN-ɣ^+^ (*p* = 0.031); 27% versus 19% TNF- α^+^ (*p* = 0.031)); 15% versus 12% IL-2^+^ (*p* = 0.031) (Figure 4A). The frequencies of TNF-α^+^ and CD154^+^ CD4^+^ T cells responding to “Omicron CD4^+^ pool” was significantly lower in T-cell lines obtained from vaccinees compared with convalescent subjects (TNF-α: median of, respectively, 5.3% versus 19% positive cells, *p* = 0.042; CD154: 13% versus 24%, *p* = 0.042). Moreover, the decrease in response from the “WT CD4^+^ pool” to the “Omicron CD4^+^ pool” was more prominent in vaccinees than in convalescent subjects (0.40- versus 0.72-fold change in percentage of IFN-ɣ^+^/CD4^+^ T cells, *p* = 0.056; 0.41- versus 0.77-fold change in percentage of TNF-α^+^/CD4^+^ T cells, *p* = 0.056; 0.39- versus 0.67-fold change in percentage of IL-2^+^/CD4^+^ T cells, *p* = 0.073; 0.41- versus 0.70-fold change in percentage of CD154^+^/CD4^+^ T cells, *p* = 0.042).

Additionally, the cytokine polyfunctionality of the T-cell lines responding to the “Omicron CD4^+^ pool” was often reduced compared with the reference “WT CD4^+^ pool”, especially in vaccinees (Figure 4B). In line with a decreased cytokine production, a significant decrease in the proportion of CD4^+^ T cells that expressed the activation marker CD154 was observed in response to the “Omicron CD4^+^ pool” compared with the “WT CD4^+^ pool” (in vaccinees: 13% versus 34% CD154^+^) (*p* = 0.0020); in convalescent subjects: 24% versus 40% CD154^+^ (*p* = 0.031)) (Figure 4A). Stimulation with the selected promiscuous helper epitopes mainly induced CD4^+^ and not CD8^+^ T cells (Supplementary Figure S1).

Taken together, these results indicate that a pool of 20 selected promiscuous helper epitopes of WT spike protein was abundantly recognized by the T-cell lines of vaccinated and previously infected individuals, while T-cell reactivity to corresponding peptides with Omicron BA.1 mutations were significantly reduced.

### 3.5. Functional Impact of Omicron BA.1 Mutations on Individual Spike-Epitope-Specific T Cells

**Figure 5:**
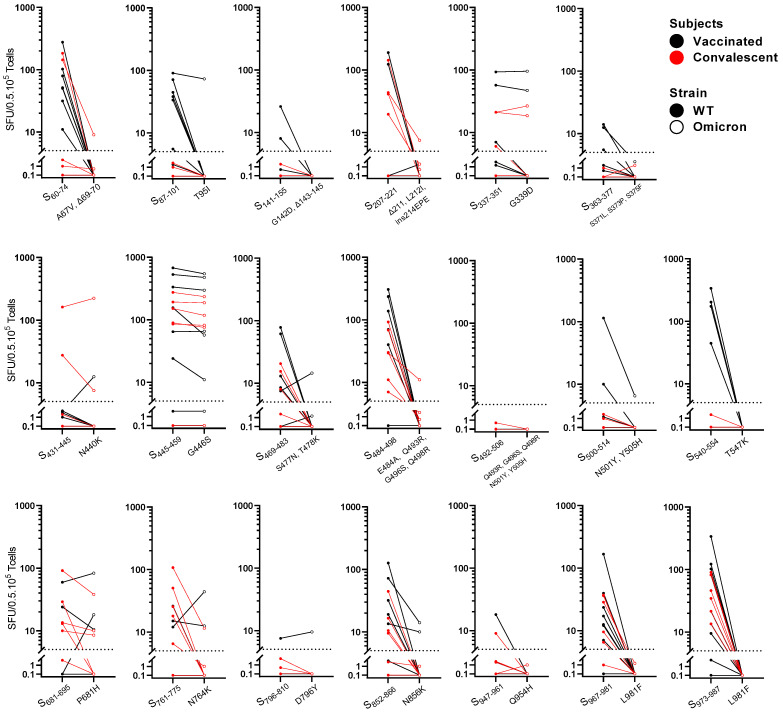
Functional impact of Omicron BA.1 mutations on individual spike-epitope-specific T cells of vaccinated and convalescent subjects. Recognition of individual CD4^+^ T-cell epitopes of spike protein by polyclonal-epitope-specific T-cell lines in IFN-ɣ ELISPOT assay. T-cell lines were generated by 14-day in vitro stimulation of PBMCs from vaccinated (black dots/lines) and convalescent individuals (red dots/lines) with a pool of selected CD4^+^ T-cell epitope candidates from the D614G wild-type (WT) strain (“WT CD4^+^ pool”) in the presence of IL-2. Subsequently, cells were stimulated for 24 h with 20 individual CD4^+^ T-cell epitopes of spike protein of WT strain versus corresponding sequences of the Omicron BA.1 variant. Each pair of dots connected with line represents SARS-CoV-2-specific IFN-ɣ^+^ T-cell response of one subject responding to an individual WT (closed dots) versus Omicron BA.1 peptide (open dots). On the *Y*-axis each individual peptide pair is indicated as location of first and last amino acid position within WT spike protein (S), whereas the corresponding Omicron BA.1 sequence is indicated by the location(s) of the amino acid residue(s) that changed compared with the WT sequence. Dotted line indicates threshold for IFN-ɣ-positive responses. SFU—spot forming units.

In order to identify which epitopes were responsible for the reduced response observed against the “Omicron CD4^+^ pool”, the reactivity of the T-cell lines was tested against the individual epitopes in an IFN-ɣ^+^ ELISPOT. In the primed T-cell lines of various previously vaccinated or convalescent subjects, good-recall T-cell responses were found against most of the selected epitopes, endorsing the good immunogenicity of these CD4^+^ T-cell epitopes and indicating that they may be considered immunodominant. Furthermore, T-cell analysis at the epitope level revealed that in the majority of cases a strong response to the CD4^+^ T-cell epitopes of WT spike coincide with a clear reduction in IFN-ɣ^+^ response against the corresponding Omicron BA.1 peptides (Figure 5). An almost complete abrogation of the IFN-ɣ^+^ T-cell response was observed for the following epitopes: S_60–74_, S_87–101_ (only vaccinees), S_207–221_, S_469–483_, S_484–498_, S_540–554_ (only vaccinees), S_761–775_ (mainly convalescent subjects), S_852–866_, S_967–981_, and S_973–987_. For various epitopes, i.e., S_337–351_, S_445–459_, and S_681–695_, if a responsiveness was found against WT epitopes, this responsiveness was mostly preserved for the epitopes with Omicron BA.1 mutations. Hardly any responsiveness was observed to epitopes S_141–155_, S_363–377_, S_492–506_, S_796–810_, and S_947–961_, while occasionally a good T-cell response was observed with epitopes S_431–445_ and S_500–514_. WT spike epitopes that showed a good IFN-ɣ T-cell response more often showed a completely abolished response when harboring > 1 mutation. In summary, an impaired epitope-specific T-cell response to Omicron spike compared with WT spike could be attributed to a total of 16 amino acid changes in the spike of the Omicron BA.1 variant: 1—A67V; 2—Δ69–70 (S_60–74_); 3—T95I (S_87–101_); 4—Δ211; 5—L212I; 6—214EPEins (S_207–221_); 7—S477N; 8—T478K (S_469–483_); 9—E484A; 10—Q493R; 11—G496S; 12—Q498R (S_484–498_); 13—T547K (S_540–554_); 14—N764K (S_761–775_); 15—N856K (S_852–866_); 16—L981F (S_967–981_ and S_973–987_).

### 3.6. Reduced Cytokine Responses to Individual Spike Epitopes Harboring Omicron BA.1 Mutations

**Figure 6:**
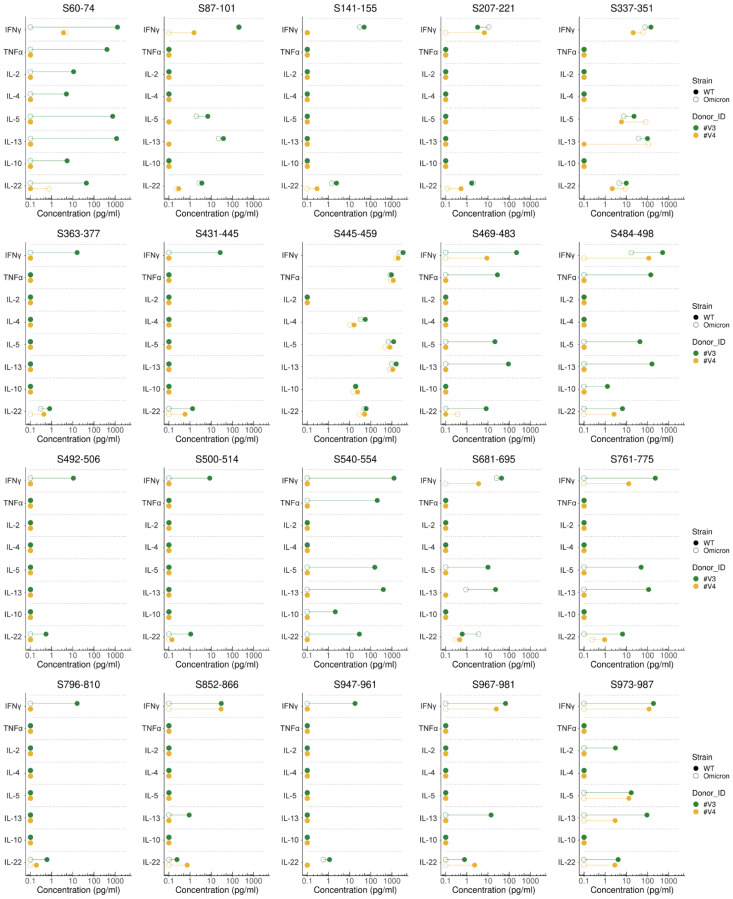
Reduced cytokine responses to the individual spike epitopes harboring Omicron BA.1 mutations. T-cell lines were generated by 14-day in vitro stimulation of PBMCs with a pool of selected CD4^+^ T-cell epitope candidates from the D614G wild-type (WT) strain (“WT CD4^+^ pool”) in the presence of IL-2. T-cell lines from two vaccinees, i.e., donor #V3 (orange) and donor #V4 (dark green), were analyzed for secretion of various cytokines detected using a flowcytometric assay. Lollipop plots show concentrations of different cytokines measured in T-cell line supernatants after stimulation of T cells with individual peptides of spike protein of D614G wild-type (WT) strain (closed dots) and corresponding Omicron BA.1 variant peptides (open dots). Differences between the response to an individual WT and a corresponding Omicron peptide are presented by a connecting line. Above each plot, the location of first and last amino acid position within WT spike protein (S) of the peptide used for stimulation are presented. Clearly reduced cytokine responses to the spike epitopes harboring Omicron mutations compared with the WT strain were observed for the following epitopes: S60–74, S469–483, S484–498, S540–554, S761–775, S967–981, and S973–987.

A comprehensive analysis of the WT-spike-enriched T-cell lines from two vaccinees and two convalescent individuals was performed to quantifiably measure release of various cytokines in supernatants upon restimulation with the individual CD4^+^ T-cell epitopes. This makes it possible to investigate whether different spike-specific T helper cell subsets are activated within the T-cell lines of vaccinees or convalescent subjects based on their signature cytokine profiles. Apart from the moderate–high levels of IFN-ɣ, IL-22 was often produced by epitope-specific T cells within the polyclonal T-cell lines obtained from the two vaccinees. In addition, IL-5 and IL-13 were produced after restimulation with various spike epitopes, and occasionally TNF-α and/or IL-2 was produced. Interestingly, only upon stimulation with the immunodominant epitope S445–459 was production of IL-4 and IL-10 observed. Clearly reduced cytokine responses to the spike epitopes harboring Omicron mutations were observed against epitopes S60–74, S469–483, S484–498, S540–554, S761–775, S967–981, and S973–987 (Figure 6). In contrast, T-cell lines from the two convalescent individuals generally showed low–moderate amounts of IFN-ɣ secretion and hardly any other cytokines were produced upon stimulation with the different helper epitopes (Supplementary Figure S2). Production of IL-6, IL-9, IL17A, and IL-17F were not detected in any of the T-cell lines after stimulation with the single peptides (data not shown). Additionally, in the T-cell lines of the two convalescent subjects, lower IFN-ɣ concentrations were generally observed after stimulation with the Omicron BA.1 peptides, although this was not the case with all epitopes.

## 4. Discussion

In the present study, immunodominant nonconserved CD4^+^ T-cell epitope regions of SARS-CoV-2 spike protein were identified that showed partial or complete loss of pre-existing T cell responsiveness due to Omicron BA.1 mutations. CD4^+^ T cells were shown to dominate the spike-specific T-cell response after SARS-CoV-2 infection [18,27,28]. A critical role for CD4^+^ T helper cells in the generation of effective neutralizing antibody responses to SARS-CoV-2 is clear [24,27]. In addition, CD4^+^ T cells may have a direct antiviral effect through cytokine secretion or killing of infected cells [24]. The importance of CD4^+^ T cells is further underlined by the fact that critically ill patients are reported to exhibit qualitatively impaired SARS-CoV-2-specific CD4^+^ T-cell responses [35]. Here, we investigated the potential impact of mutations associated with Omicron BA.1 variant on reactivity of pre-existing CD4^+^ T cells of prior mRNA-vaccinated or convalescent individuals. Various studies investigating the SARS-CoV-2-specific T-cell cross-recognition of the Omicron BA.1 variant have been restricted to sequence comparison in combination with in silico prediction of peptide–HLA binding [25,26]. One of these studies revealed that 28% of 167 CD4^+^ T-cell epitopes and 14% of 224 CD8^+^ T-cell epitopes of spike protein available at IEDB comprise at least one position harboring an Omicron BA.1 mutation in its amino acid sequence [26]. The functional T-cell response against the Omicron BA.1 variant has been investigated by others using overlapping peptide pools spanning the whole spike protein [13,19,21,36,37]. In general, these studies show that overall memory T-cell responses of prior-vaccinated and convalescent individuals with ancestral SARS-CoV-2 are relatively well preserved against Omicron BA.1 whole spike protein, which is in agreement with our ex vivo IFN-ɣ ELISPOT data. Remarkably, in PBMCs of convalescent subjects that were taken 6–8 weeks post symptom onset, we even observed a slightly higher frequency of IFN-ɣ^+^ T cells responding to overlapping peptides spanning the whole spike protein of the Omicron BA.1 variant compared with spike of the ancestral D614G wild-type (WT) (Figure 2A). This suggests that the spike-specific T-cell epitope repertoires induced after infection differ from those elicited upon mRNA vaccination. In another study, it was shown that spike-derived epitopes were not dominantly targeted in convalescent individuals compared with non-spike epitopes [38]. Therefore, immunogenic proteins other than spike, which are not addressed in this study, with fewer mutations in the Omicron BA.1 variant, may contribute to conserved-memory T-cell responses in naturally infected individuals.

By using overlapping peptides spanning the whole spike protein, it is impossible to assess the functional impact on T-cell recognition of the specific Omicron BA.1 mutations present in individual epitopes. To be able to identify specific epitope mismatches, we selected for CD4^+^ the T-cell epitope candidates of the WT spike protein (“WT CD4^+^ pool”) carrying at least one Omicron BA.1 mutation. Another selection criterion was that the epitope candidates were predicted to be restricted by a large number of common HLA-II alleles. An advantage of these more universal T helper cell epitopes is that they can be more prevalently recognized by the T cells of the hum an population. Although the direct ex vivo recall T-cell responses to these selected CD4^+^ T-cell epitope pools appeared to be generally low, a clear IFN-ɣ^+^ T-cell response to the “WT CD4^+^ pool” was found in the PBMCs of two vaccinated individuals that showed a significantly reduced cross-reactivity to the corresponding Omicron BA.1 peptide sequences (Figure 2B). T-cell lines generated by in vitro stimulation with the “WT CD4^+^ pool” showed abundant responses to this WT peptide pool in vaccinees and convalescent individuals, while T-cell reactivity was significantly reduced in the pool with corresponding Omicron BA.1 peptide sequences (Figure 3).

At the individual epitope level, T-cell lines revealed strong IFN-ɣ^+^ responses against most of the 20 selected WT spike CD4^+^ T-cell epitopes in the majority of vaccinees and prior-infected subjects, confirming good immunogenicity and broad HLA-II restriction of these spike epitopes (Figure 5). Apart from IFN-ɣ, IL-22, and occasionally TNF-α, IL-5 and IL-13 were also produced by T-cell lines of the two tested vaccinees, indicating that vaccination induces a mixed Th1 and Th2 CD4^+^ T-cell response against the spike epitopes (Figure 6). In contrast, T-cell lines from the two convalescent individuals generally showed hardly any cytokine secretion, apart from IFN-ɣ (Supplementary Figure S2). Furthermore, the T-cell lines of vaccinees and convalescent individuals show a different pattern of immunodominance of the spike epitopes (Figure 5), as other epitopes sometimes lead to a good response in vaccinated subjects rather than in convalescent subjects and vice versa. This implies the induction of different T-cell epitope repertoires after mRNA vaccination and infection.

An impaired IFN-ɣ^+^ T-cell reactivity against the Omicron BA.1 peptide counterparts was found in as many as 15 out of the 20 WT spike epitopes (Figure 5). The reduced T-cell response to the Omicron BA.1 peptides was, on average, more significant in prior-vaccinated subjects than in prior-infected subjects. Several of the selected CD4^+^ T-cell epitopes harboring Omicron BA.1 mutations showed almost a complete abrogation of the IFN-ɣ^+^ T-cell response compared with the WT ancestral sequences, such as S_60–74_, S_87–101_ (only vaccinees), S_207–221_, S_469–483_, S_484–498_, S_540–554_ (only vaccinees), S_761–775_ (mainly convalescent subjects), S_852–866_, S_967–981_, and S_973–987_. It is noteworthy that the sequence of S_89–97_ includes the confirmed HLA-A*03:01-, HLA-A*11:01-, or HLA-A*68:01-restricted CD8^+^ T-cell epitope GVYFASTEK (IEDB ID 1037798, 1037940, and 1039976) [25,39,40,41], which could also be affected by the Omicron BA.1 mutation T95I. On the other hand, a well-preserved responsiveness to the following Omicron BA.1 peptides was observed: S_337–351_, S_445–459_, and S_681–695_. In another study, T cells were found in 50% of the tested donors that were specific for 20-mer peptide S_446–465_, which includes our S_445–459_ epitope sequence [42], confirming the immunodominance of this epitope. Verhagen et al. also identified S_445–459_ and S_449–463_ as immunodominant epitopes [43]. Strikingly, the Omicron sub-lineages BA.4 and BA.5 carry an extra mutation (L452R) within this epitope region that could possibly abolish the T-cell cross-reactivity. Moreover, this L452R mutation is also present in the earlier SARS-CoV-2 variants, i.e., Kappa (B.1.617.1) and Delta (B.1.617.2) variants.

Loss of T-cell recognition may be caused by disruption of antigen processing, impaired binding of HLA-II to peptides, or impaired T-cell receptor (TCR) recognition of the HLA–peptide complex. Reduced peptide–HLA-II binding can occur when a mutation affects (one of) the anchor residues. Since each individual HLA-II allotype exhibits its own peptide-binding motif with its specific anchor positions, it is difficult to determine which mutations do or do not involve an anchor position for our selection of epitope candidates with broad HLA-II restriction potential. This is further complicated by the occurrence of sometimes multiple mutations/deletions or an insert within a single T-cell epitope. Interestingly, instead of an impaired HLA-II binding, an even better HLA-II binding prediction score was observed for the Omicron BA.1 variant sequence of S_761–775_ (also present in Omicron BA.4/BA.5 variants) and S_852–866_ (Figure 1). Yet, these variant epitopes showed a reduced T-cell response, most likely caused by loss of TCR recognition. Implementation of these Omicron sequences in an updated vaccine may yield new immunodominant epitopes that contribute to protection against Omicron variants.

This study had some limitations, as follows: The two groups of vaccinated (*n* = 10) and convalescent subjects (*n* = 6) used in this comprehensive T-cell analysis at epitope level were small. To mitigate strong individual donor effects on group responses, group variables that may likely to affect T-cell (cross-)reactivity were kept as constant as possible. All vaccinees received the mRNA-based BNT162b2 vaccine in the same schedule (2 doses with a 35-day interval). The group of convalescent persons were all infected during March–May 2020. In addition, the timepoint for sampling was uniform for subjects of each group (28 days after their second vaccine dose, and 6–8 weeks post symptom onset, respectively). The age range for both groups was 18–51 years, and they comprised 50% males and 50% females. Furthermore, the assays used in our study tested peptide-based responses rather than the responses of the native viral antigen that will occur in vivo. However, mutations in the virus—even outside the epitope region—might influence antigen uptake, antigen processing, and/or epitope presentation by antigen-presenting cells.

## 5. Conclusions

Despite the > 30 amino acid changes in Omicron BA.1 variant spike protein, the overall memory CD4^+^ T-cell response primed by the 1273-amino-acid-long ancestral spike protein in prior-vaccinated and naturally infected persons seems preserved in the small group of subjects of this study. In addition, other immunogenic SARS-CoV-2 proteins with less mutations in the Omicron BA.1 variant may also contribute to preserved memory T-cell responses in persons that were naturally infected with previously circulating SARS-CoV-2 variants. However, there is also a clear loss of memory CD4^+^ T-cell reactivity to immunodominant epitopes across the Omicron spike protein due to mutations. Various mutations in spike proteins also occur in the newer Omicron BA.2, BA.3, BA.4, and BA.5 sub-lineages. Monitoring such specific mutations in the global virus population could help to identify the extent to which SARS-CoV-2 variants could escape from preexisting CD4^+^ T cell memory and be of concern. Furthermore, variant-adapted vaccines covering relevant variable sequences of spike and/or additional sequences of other immunogenic proteins may be required to optimally broaden the immune responses toward emerging SARS-CoV-2 viruses.

## Figures and Tables

**Table 1:** List of selected CD4^+^ T-cell epitope candidates of spike protein of the wild-type reference strain having mutations in the corresponding Omicron BA.1 variant sequence.

	Pos	WT CD4^+^ Pool	Omicron BA.1 Mutation	Omicron CD4^+^ Pool
1	S_60–74_	SNVTWFHAIHVSGTN	A67V, Δ69–70	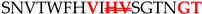
2	S_87–101_	NDGVYFASTEKSNII	T95I	NDGVYFAS**I**EKSNII
3	S_141–155_	LGVYYHKNNKSWMES	G142D, Δ143–145	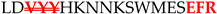
4	S_207–221_	HTPINLVRDLPQGFS	Δ211, L212I, 214EPEins	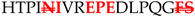
5	S_337–351_	PFGEVFNATRFASVY	G339D	PF**D**EVFNATRFASVY
6	S_363–377_	ADYSVLYNSASFSTF	S371L, S373P, S375F	ADYSVLYN**L**A**P**F**F**TF
7	S_431–445_	GCVIAWNSNNLDSKV	N440K	GCVIAWNSN**K**LDSKV
8	S_445–459_	VGGNYNYLYRLFRKS	G446S	V**S**GNYNYLYRLFRKS
9	S_469–483_	STEIYQAGSTPCNGV	S477N, T478K	STEIYQAG**NK**PCNGV
10	S_484–498_	EGFNCYFPLQSYGFQ	E484A, Q493R, G496S, Q498R	**A**GFNCYFPL**R**SY**S**F**R**
11	S_492–506_	LQSYGFQPTNGVGYQ	Q493R, G496S, Q498R, N501Y, Y505H	L**R**SY**S**F**R**PT**Y**GVG**H**Q
12	S_500–514_	TNGVGYQPYRVVVLS	N501Y, Y505H	T**Y**GVG**H**QPYRVVVLS
13	S_540–554_	NFNFNGLTGTGVLTE	T547K	NFNFNGL**K**GTGVLTE
14	S_681–695_	PRRARSVASQSIIAY	P681H	**H**RRARSVASQSIIAY
15	S_761–775_	TQLNRALTGIAVEQD	N764K	TQL**K**RALTGIAVEQD
16	S_796–810_	DFGGFNFSQILPDPS	D796Y	**Y**FGGFNFSQILPDPS
17	S_852–866_	AQKFNGLTVLPPLLT	N856K	AQKF**K**GLTVLPPLLT
18	S_947–961_	KLQDVVNQNAQALNT	Q954H	KLQDVVN**H**NAQALNT
19	S_967–981_	SSNFGAISSVLNDIL	L981F	SSNFGAISSVLNDI**F** ^a^
20	S_973–987_	ISSVLNDILSRLDKV	L981F	ISSVLNDI**F**SRLDKV

^a^ Notably, Omicron BA.1 mutation N969K was not included in the study, and is therefore missing in the used peptides. The location of the peptides (all 15-mers) are shown as the position of the first and last amino acid of the peptide within spike protein. Amino acid mutations in Omicron BA.1 sequences are shown as bold red font. Abbreviations: WT—D614G wild-type reference strain; Omicron—Omicron BA.1 strain.

## Data Availability

The raw data supporting the conclusions of this article will be made available by the authors upon request, with consideration of the participants’ privacy and ethical rights.

## Supplementary Materials

The following supporting information can be downloaded at: https://www.mdpi.com/article/10.3390/v14071570/s1, Figure S1: Mainly CD4^+^ and not CD8^+^ T cells were activated upon stimulation with selected CD4^+^ T-cell epitope pools, Figure S2: Cytokine production of T-cell lines from two convalescent subjects in response to individual spike epitopes harboring Omicron BA.1 mutations, Table S1: Selected CD4^+^ T-cell epitope candidates of the spike protein of the D614G wild-type reference strain with best matching epitope sequence available from IEDB.

## References

[1] Classification of Omicron (B.1.1.529): SARS-CoV-2 Variant of Concern. https://www.who.int/.

[2] Garcia-Beltran W.F., St Denis K.J., Hoelzemer A., Lam E.C., Nitido A.D., Sheehan M.L., Berrios C., Ofoman O., Chang C.C., Hauser B.M. (2022). mRNA-based COVID-19 vaccine boosters induce neutralizing immunity against SARS-CoV-2 Omicron variant. Cell.

[3] Hoffmann M., Kruger N., Schulz S., Cossmann A., Rocha C., Kempf A., Nehlmeier I., Graichen L., Moldenhauer A.S., Winkler M.S. (2022). The Omicron variant is highly resistant against antibody-mediated neutralization: Implications for control of the COVID-19 pandemic. Cell.

[4] Liu L., Iketani S., Guo Y., Chan J.F., Wang M., Liu L., Luo Y., Chu H., Huang Y., Nair M.S. (2022). Striking antibody evasion manifested by the Omicron variant of SARS-CoV-2. Nature.

[5] Planas D., Saunders N., Maes P., Guivel-Benhassine F., Planchais C., Buchrieser J., Bolland W.H., Porrot F., Staropoli I., Lemoine F. (2022). Considerable escape of SARS-CoV-2 Omicron to antibody neutralization. Nature.

[6] Schmidt F., Muecksch F., Weisblum Y., Da Silva J., Bednarski E., Cho A., Wang Z., Gaebler C., Caskey M., Nussenzweig M.C. (2022). Plasma Neutralization of the SARS-CoV-2 Omicron Variant. N. Engl. J. Med..

[7] Karim S.S.A., Karim Q.A. (2021). Omicron SARS-CoV-2 variant: A new chapter in the COVID-19 pandemic. Lancet.

[8] Baden L.R., El Sahly H.M., Essink B., Kotloff K., Frey S., Novak R., Diemert D., Spector S.A., Rouphael N., Creech C.B. (2021). Efficacy and Safety of the mRNA-1273 SARS-CoV-2 Vaccine. N. Engl. J. Med..

[9] Polack F.P., Thomas S.J., Kitchin N., Absalon J., Gurtman A., Lockhart S., Perez J.L., Perez Marc G., Moreira E.D., Zerbini C. (2020). Safety and Efficacy of the BNT162b2 mRNA COVID-19 Vaccine. N. Engl. J. Med..

[10] Sadoff J., Gray G., Vandebosch A., Cardenas V., Shukarev G., Grinsztejn B., Goepfert P.A., Truyers C., Fennema H., Spiessens B. (2021). Safety and Efficacy of Single-Dose Ad26.COV2.S Vaccine against COVID-19. N. Engl. J. Med..

[11] Voysey M., Clemens S.A.C., Madhi S.A., Weckx L.Y., Folegatti P.M., Aley P.K., Angus B., Baillie V.L., Barnabas S.L., Bhorat Q.E. (2021). Safety and efficacy of the ChAdOx1 nCoV-19 vaccine (AZD1222) against SARS-CoV-2: An interim analysis of four randomised controlled trials in Brazil, South Africa, and the UK. Lancet.

[12] Heath P.T., Galiza E.P., Baxter D.N., Boffito M., Browne D., Burns F., Chadwick D.R., Clark R., Cosgrove C., Galloway J. (2021). Safety and Efficacy of NVX-CoV2373 COVID-19 Vaccine. N. Engl. J. Med..

[13] GeurtsvanKessel C.H., Geers D., Schmitz K.S., Mykytyn A.Z., Lamers M.M., Bogers S., Scherbeijn S., Gommers L., Sablerolles R.S.G., Nieuwkoop N.N. (2022). Divergent SARS-CoV-2 Omicron-reactive T- and B cell responses in COVID-19 vaccine recipients. Sci. Immunol..

[14] Bertoletti A., Le Bert N., Qui M., Tan A.T. (2021). SARS-CoV-2-specific T cells in infection and vaccination. Cell Mol. Immunol..

[15] Tan A.T., Linster M., Tan C.W., Le Bert N., Chia W.N., Kunasegaran K., Zhuang Y., Tham C.Y.L., Chia A., Smith G.J.D. (2021). Early induction of functional SARS-CoV-2-specific T cells associates with rapid viral clearance and mild disease in COVID-19 patients. Cell Rep..

[16] Moderbacher C.R., Ramirez S.I., Dan J.M., Grifoni A., Hastie K.M., Weiskopf D., Belanger S., Abbott R.K., Kim C., Choi J. (2020). Antigen-Specific Adaptive Immunity to SARS-CoV-2 in Acute COVID-19 and Associations with Age and Disease Severity. Cell.

[17] Sekine T., Perez-Potti A., Rivera-Ballesteros O., Stralin K., Gorin J.B., Olsson A., Llewellyn-Lacey S., Kamal H., Bogdanovic G., Muschiol S. (2020). Robust T Cell Immunity in Convalescent Individuals with Asymptomatic or Mild COVID-19. Cell.

[18] Moss P. (2022). The T cell immune response against SARS-CoV-2. Nat. Immunol..

[19] Tarke A., Coelho C.H., Zhang Z., Dan J.M., Yu E.D., Methot N., Bloom N.I., Goodwin B., Phillips E., Mallal S. (2022). SARS-CoV-2 vaccination induces immunological T cell memory able to cross-recognize variants from Alpha to Omicron. Cell.

[20] Bange E.M., Han N.A., Wileyto P., Kim J.Y., Gouma S., Robinson J., Greenplate A.R., Hwee M.A., Porterfield F., Owoyemi O. (2021). CD8^+^ T cells contribute to survival in patients with COVID-19 and hematologic cancer. Nat. Med..

[21] Naranbhai V., Nathan A., Kaseke C., Berrios C., Khatri A., Choi S., Getz M.A., Tano-Menka R., Ofoman O., Gayton A. (2022). T cell reactivity to the SARS-CoV-2 Omicron variant is preserved in most but not all individuals. Cell.

[22] Gao Y., Cai C., Grifoni A., Muller T.R., Niessl J., Olofsson A., Humbert M., Hansson L., Osterborg A., Bergman P. (2022). Ancestral SARS-CoV-2-specific T cells cross-recognize the Omicron variant. Nat. Med..

[23] Tarke A., Sidney J., Methot N., Yu E.D., Zhang Y., Dan J.M., Goodwin B., Rubiro P., Sutherland A., Wang E. (2021). Impact of SARS-CoV-2 variants on the total CD4^+^ and CD8^+^ T cell reactivity in infected or vaccinated individuals. Cell Rep. Med..

[24] Kent S.J., Khoury D.S., Reynaldi A., Juno J.A., Wheatley A.K., Stadler E., John Wherry E., Triccas J., Sasson S.C., Cromer D. (2022). Disentangling the relative importance of T cell responses in COVID-19: Leading actors or supporting cast?. Nat. Rev. Immunol..

[25] Redd A.D., Nardin A., Kared H., Bloch E.M., Abel B., Pekosz A., Laeyendecker O., Fehlings M., Quinn T.C., Tobian A.A.R. (2022). Minimal Crossover between Mutations Associated with Omicron Variant of SARS-CoV-2 and CD8^+^ T-Cell Epitopes Identified in COVID-19 Convalescent Individuals. mBio.

[26] Ahmed S.F., Quadeer A.A., McKay M.R. (2022). SARS-CoV-2 T Cell Responses Elicited by COVID-19 Vaccines or Infection Are Expected to Remain Robust against Omicron. Viruses.

[27] Juno J.A., Tan H.X., Lee W.S., Reynaldi A., Kelly H.G., Wragg K., Esterbauer R., Kent H.E., Batten C.J., Mordant F.L. (2020). Humoral and circulating follicular helper T cell responses in recovered patients with COVID-19. Nat. Med..

[28] Kaaijk P., Pimentel V.O., Emmelot M.E., Poelen M., Cevirgel A., Schepp R.M., den Hartog G., Reukers D.F.M., Beckers L., van Beek J. (2022). Children and Adults with Mild COVID-19: Dynamics of the Memory T Cell Response up to 10 Months. Front. Immunol..

[29] McKinney D.M., Southwood S., Hinz D., Oseroff C., Arlehamn C.S., Schulten V., Taplitz R., Broide D., Hanekom W.A., Scriba T.J. (2013). A strategy to determine HLA class II restriction broadly covering the DR, DP, and DQ allelic variants most commonly expressed in the general population. Immunogenetics.

[30] Reukers D.F.M., van Boven M., Meijer A., Rots N., Reusken C., Roof I., van Gageldonk-Lafeber A.B., van der Hoek W., van den Hof S. (2022). High Infection Secondary Attack Rates of Severe Acute Respiratory Syndrome Coronavirus 2 in Dutch Households Revealed by Dense Sampling. Clin. Infect. Dis..

[31] van den Hoogen L.L., Smits G., van Hagen C.C.E., Wong D., Vos E.R.A., van Boven M., de Melker H.E., van Vliet J., Kuijer M., Woudstra L. (2022). Seropositivity to Nucleoprotein to detect mild and asymptomatic SARS-CoV-2 infections: A complementary tool to detect breakthrough infections after COVID-19 vaccination?. Vaccine.

[32] ECDC (2021). Implications of the Emergence and Spread of the SARS-CoV-2 B.1.1. 529 Variant of Concern (Omicron) for the EU/EEA. Threath Assessment Brief. https://www.ecdc.europa.eu/sites/default/files/documents/Implications-emergence-spread-SARS-CoV-2%20B.1.1.529-variant-concern-Omicron-for-the-EU-EEA-Nov2021.pdf.

[33] Reynisson B., Barra C., Kaabinejadian S., Hildebrand W.H., Peters B., Nielsen M. (2020). Improved Prediction of MHC II Antigen Presentation through Integration and Motif Deconvolution of Mass Spectrometry MHC Eluted Ligand Data. J. Proteome Res..

[34] Vita R., Mahajan S., Overton J.A., Dhanda S.K., Martini S., Cantrell J.R., Wheeler D.K., Sette A., Peters B. (2019). The Immune Epitope Database (IEDB): 2018 update. Nucleic Acids Res..

[35] Oja A.E., Saris A., Ghandour C.A., Kragten N.A.M., Hogema B.M., Nossent E.J., Heunks L.M.A., Cuvalay S., Slot E., Linty F. (2020). Divergent SARS-CoV-2-specific T- and B-cell responses in severe but not mild COVID-19 patients. Eur. J. Immunol..

[36] Choi S.J., Kim D.U., Noh J.Y., Kim S., Park S.H., Jeong H.W., Shin E.C. (2022). T cell epitopes in SARS-CoV-2 proteins are substantially conserved in the Omicron variant. Cell Mol. Immunol..

[37] Keeton R., Tincho M.B., Ngomti A., Baguma R., Benede N., Suzuki A., Khan K., Cele S., Bernstein M., Karim F. (2022). T cell responses to SARS-CoV-2 spike cross-recognize Omicron. Nature.

[38] Lang-Meli J., Luxenburger H., Wild K., Karl V., Oberhardt V., Salimi Alizei E., Graeser A., Reinscheid M., Roehlen N., Reeg D.B. (2022). SARS-CoV-2-specific T-cell epitope repertoire in convalescent and mRNA-vaccinated individuals. Nat. Microbiol..

[39] Tarke A., Sidney J., Kidd C.K., Dan J.M., Ramirez S.I., Yu E.D., Mateus J., da Silva Antunes R., Moore E., Rubiro P. (2021). Comprehensive analysis of T cell immunodominance and immunoprevalence of SARS-CoV-2 epitopes in COVID-19 cases. Cell Rep. Med..

[40] Wagner K.I., Mateyka L.M., Jarosch S., Grass V., Weber S., Schober K., Hammel M., Burrell T., Kalali B., Poppert H. (2022). Recruitment of highly cytotoxic CD8^+^ T cell receptors in mild SARS-CoV-2 infection. Cell Rep..

[41] Kared H., Redd A.D., Bloch E.M., Bonny T.S., Sumatoh H., Kairi F., Carbajo D., Abel B., Newell E.W., Bettinotti M.P. (2021). SARS-CoV-2-specific CD8^+^ T cell responses in convalescent COVID-19 individuals. J. Clin. Investig..

[42] Low J.S., Vaqueirinho D., Mele F., Foglierini M., Jerak J., Perotti M., Jarrossay D., Jovic S., Perez L., Cacciatore R. (2021). Clonal analysis of immunodominance and cross-reactivity of the CD4 T cell response to SARS-CoV-2. Science.

[43] Verhagen J., van der Meijden E.D., Lang V., Kremer A.E., Volkl S., Mackensen A., Aigner M., Kremer A.N. (2021). Human CD4^+^ T cells specific for dominant epitopes of SARS-CoV-2 Spike and Nucleocapsid proteins with therapeutic potential. Clin. Exp. Immunol..

