# Supplementary material for: Omicron BA.1 Mutations in SARS-CoV-2 Spike Lead to Reduced T-Cell Response in Vaccinated and Convalescent Individuals
Source: Viruses. 2022 Jul 19;14(7):1570. doi: 10.3390/v14071570 (PMC9318964; doi:10.3390/v14071570)
Supplement: Supplementary file 1 [file viruses-14-01570-s001.zip › viruses-1804739-supplementary.pdf]

Supplementary Materials

Supplementary Figures

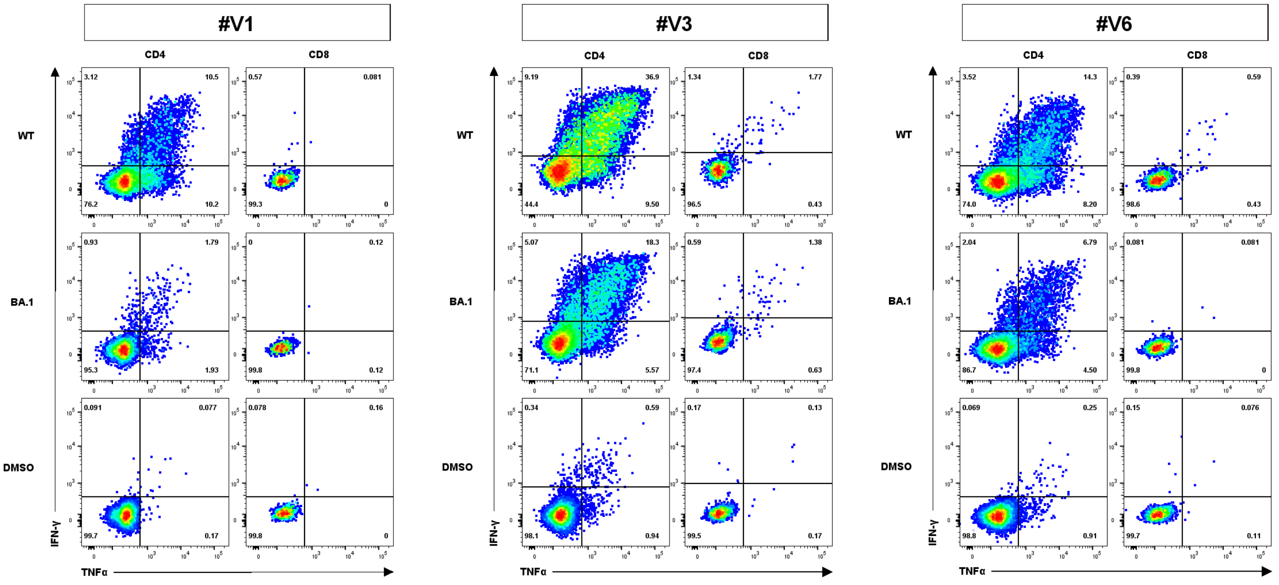

Figure S1. Mainly CD4$^+$ and not CD8$^+$ T cells were activated upon stimulation with selected CD4$^+$ T cell epitope pools

FACS plots showing intracellular cytokine staining of IFN-γ (X-axis) and TNF-α (Y-axis) of stimulated T cell lines of three representative vaccinated subjects (#V1 (left panel), #V3 (middle panel), and #V6 (right panel)). For each of the three subjects, T cells gated on CD4$^+$ expression are shown in the left plots, and T cells gated on CD8$^+$ expression are presented in the right plots. From the top down, T cell lines stimulated with respectively, pool of selected CD4$^+$ T cell epitope candidates from the D614G wild-type (WT) strain ("WT CD4+ pool"), the corresponding Omicron BA.1 variant peptides ("Omicron CD4+ pool") (BA.1) and DMSO (negative control). Stimulation with the selected promiscuous helper epitopes mainly induced intracellular cytokine expression in CD4$^+$ and not CD8$^+$ T cells. Expression of intracellular cytokines was reduced in response to "Omicron CD4$^+$ pool" compared to "WT CD4$^+$ pool". Numbers indicate the percentage of cells in each quadrant.

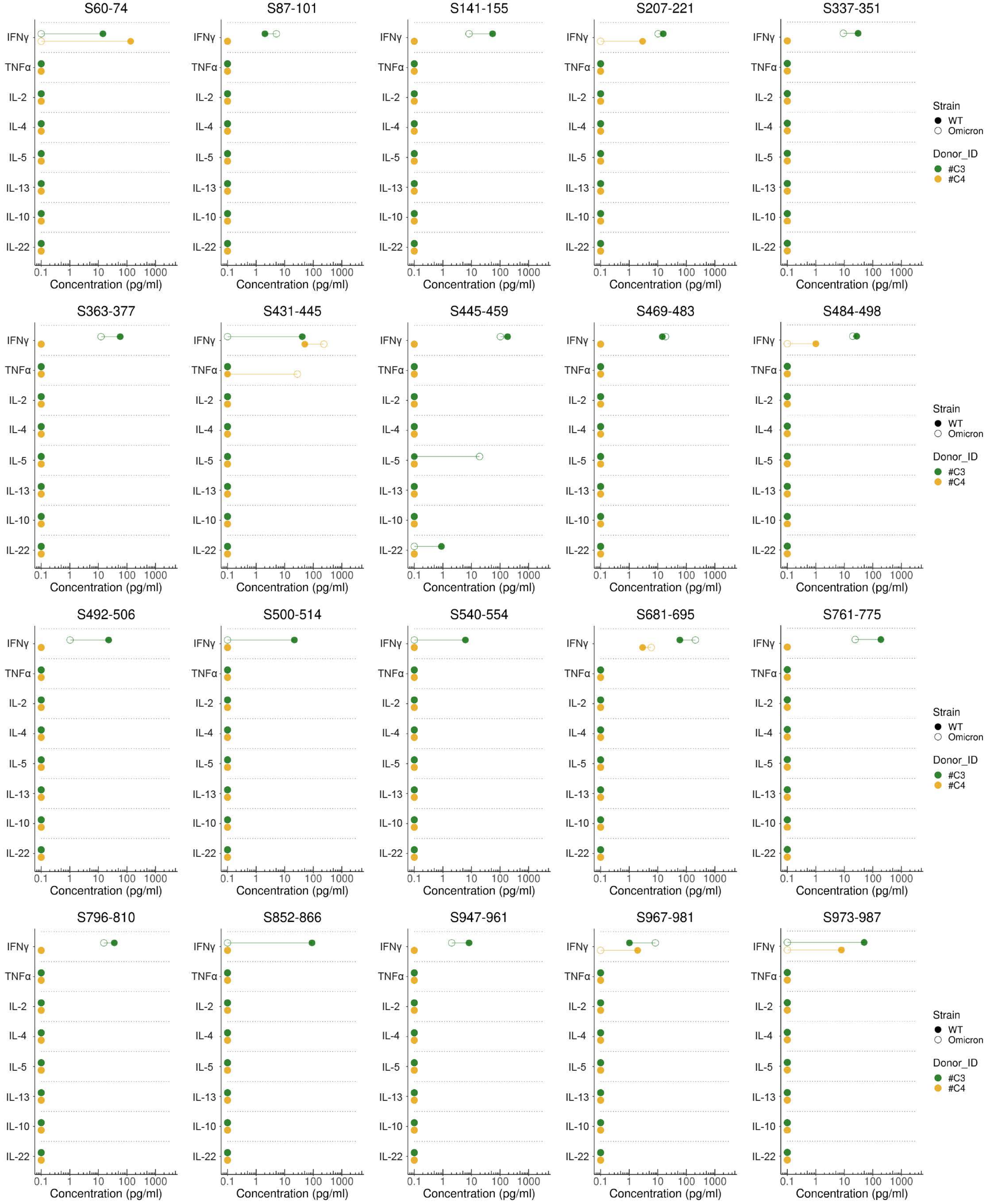
S60-74
S87-101
S141-155
S207-221
S337-351
S363-377
S431-445
S445-459
S469-483
S484-498
S492-506
S500-514
S540-554
S681-695
S761-775
S796-810
S852-866
S947-961
S967-981
S973-987
IFNγ
TNFα
IL-2
IL-4
IL-5
IL-13
IL-10
IL-22
0.1
1
10
100
1000
Concentration (pg/ml)
Strain
WT
Omicron
Donor_ID
#C3
#C4

Figure S2. Cytokine production of T cell lines from two convalescent subjects in response to individual spike epitopes harboring Omicron BA.1 mutations

T cell lines were generated by 14-days in vitro stimulation of PBMCs with a pool of selected CD4+ T cell epitope candidates from the D614G wild-type (WT) strain ("WT CD4+ pool") in the presence of IL-2. T cell lines from two convalescent subjects, i.e., donor #C3 (orange) and donor #C4 (dark green), were analyzed for secretion of various cytokines detected using a flowcytometric assay.

Lollipop plots show concentrations of different cytokines measured in T cell line supernatants after stimulation of T cells with individual peptides of spike protein of D614G wild-type (WT) strain (closed dots) and corresponding Omicron BA.1 variant peptides (open dots). Differences between the response to an individual WT and a corresponding Omicron peptide are presented by a connecting line. Above each plot, the location of first and last amino acid position within WT spike protein (S) of the peptide used for stimulation are presented. In general, lower IFN-ɣ concentrations were observed after stimulation with the Omicron BA.1 peptides, although this was not the case with all epitopes.

Table S1. Selected CD4[+] T cell epitope candidates of the spike protein of the D614G wild-type reference strain with best matching epitope sequence available from IEDB

Location peptide	WT CD4[+] spike epitopes	IEDB	IEDB epitope sequences[a]
$S_{60\text{-}74}$	SNVTWFHAIHVSGTN	1310701	**NVTWFHAIHVSGTN**G
$S_{87\text{-}101}$	NDGVYFASTEKSNII	1397173	**NDGVYFASTEKSNII**R
$S_{141\text{-}155}$	LGVYYHKNNKSWMES	1310575	**LGVYYHKNNKSWMES**
$S_{207\text{-}221}$	HTPINLVRDLPQGFS	1309123	K**HTPINLVRDLPQGF**
$S_{337\text{-}351}$	PFGEVFNATRFASVY	1310312	C**PFGEVFNATRFASV**
$S_{363\text{-}377}$	ADYSVLYNSASFSTF	1069291	CV**ADYSVLYNSASFS**
$S_{431\text{-}445}$	GCVIAWNSNNLDSKV	1310437	**GCVIAWNSNNLDSKV**
$S_{445\text{-}459}$	VGGNYNYLYRLFRKS	1073698	**VGGNYNYLYRLFRKS**
$S_{469\text{-}483}$	STEIYQAGSTPCNGV	1313689	**STEIYQAGSTPCNGV**
$S_{484\text{-}498}$	EGFNCYFPLQSYGFQ	1397221	V**EGFNCYFPLQSYGFQ**PT
$S_{492\text{-}506}$	LQSYGFQPTNGVGYQ	1397166	**LQSYGFQPTNGVGYQ**PY
$S_{500\text{-}514}$	TNGVGYQPYRVVVLS	1540449	GFQP**TNGVGYQPYRVVVLS**F
$S_{540\text{-}554}$	NFNFNGLTGTGVLTE	1069550	**FNFNGLTGTGVLTE**S
$S_{681\text{-}695}$	PRRARSVASQSIIAY	1394068	S**PRRARSVASQSIIAY**T
$S_{761\text{-}775}$	TQLNRALTGIAVEQD	1310863	**TQLNRALTGIAVEQD**
$S_{796\text{-}810}$	DFGGFNFSQILPDPS	1312421	**FGGFNFSQILPDPS**K
$S_{852\text{-}866}$	AQKFNGLTVLPPLLT	1310303	C**AQKFNGLTVLPPLL**
$S_{947\text{-}961}$	KLQDVVNQNAQALNT	1310448	G**KLQDVVNQNAQALN**
$S_{967\text{-}981}$	SSNFGAISSVLNDIL	1313359	QL**SSNFGAISSVLND**
$S_{973\text{-}987}$	ISSVLNDILSRLDKV	1312775	**ISSVLNDILSRLDKV**

[a]Identified IEDB epitope sequences of SARS-CoV-2 spike protein (UniProtKB: P0DTC2, host: human) that were tested positive in a T cell assay according to information in IEDB
The location of the peptide is shown as the position of the first and last amino acid of the peptide within the spike protein. Each matching amino acid sequence of the selected WT CD4[+] spike epitope candidates with the closest matching SARS-CoV-2 spike epitope sequence from IEDB is shown as bold character.
Abbreviations: IEDB, The Immune Epitope Database (www.iedb.org)
